# Evaluation of CDC light traps for mosquito surveillance in a malaria endemic area on the Thai-Myanmar border

**DOI:** 10.1186/s13071-015-1225-3

**Published:** 2015-12-15

**Authors:** Patchara Sriwichai, Stephan Karl, Yudthana Samung, Suchada Sumruayphol, Kirakorn Kiattibutr, Anon Payakkapol, Ivo Mueller, Guiyun Yan, Liwang Cui, Jetsumon Sattabongkot

**Affiliations:** Department of Medical Entomology, Faculty of Tropical Medicine, Mahidol University, Bangkok, Thailand; Population Health and Immunity Division, Walter and Eliza Hall Institute of Medical Research, Melbourne, Australia; Program in Public Health, University of California, Irvine, CA USA; Department of Entomology, Pennsylvania State University, University Park, PA USA; Mahidol Vivax Research Unit, Faculty of Tropical Medicine, Mahidol University, Bangkok, Thailand

**Keywords:** CDC light trap, Dry ice, CO_2_, *Anopheles*, *Culex*, *Aedes*, Malaria, Thailand

## Abstract

**Background:**

Centers for Disease Control and Prevention miniature light traps (CDC-LT) baited with CO_2_ are a routine tool for adult mosquito sampling used in entomological surveys, and for monitoring and surveillance of disease vectors. The present study was aimed at evaluating the performance of baited and unbaited CDC-LT for indoor and outdoor trapping of endemic mosquito species in northwestern Thailand.

**Methods:**

CDC-LT (*n* = 112) with and without dry ice baits were set both indoors and outdoors in 88 selected houses for stretches of 5 consecutive nights per month in 7 villages in Tha Song Yang district, Tak province between January 2011 and March 2013. Individual traps were repeatedly placed in the same location for a median of 6 (range 1–10) times. Mosquitoes were identified by morphological characteristics and classified into blood-fed, empty, male/female and gravid. Absolute mosquito numbers were converted to capture rates (i.e., mosquitoes per trap and year). Capture rates were compared using multilevel negative binomial regression to account for multiple trap placements and adjust for regional and seasonal differences.

**Results:**

A total of 6,668 mosquitoes from 9 genera were collected from 576 individual CDC-LT placements. *Culex* was the predominant captured genus (46 %), followed by anopheline mosquitoes (45 %). Overall, CO_2_ baited traps captured significantly more *Culex* (especially *Culex vishnui* Theobald) and *Anopheles* mosquitoes per unit time (adjusted capture rate ratio (aCRR) 1.64 and 1.38, respectively). *Armigeres* spp. mosquitoes were trapped in outdoor traps with significantly higher frequency (aCRR: 1.50), whereas *Aedes albopictus* (Skuse) had a tendency to be trapped more frequently indoors (aCRR: 1.89, *p* = 0.07). Furthermore, capture rate ratios between CO_2_ baited and non-baited CDC-LT were significantly influenced by seasonality and indoor vs. outdoor trap placement.

**Conclusion:**

The present study shows that CDC-LT with CO_2_ baiting capture significantly more *Culex* and *Anopheles* mosquitoes, some of which (e.g., *Cx. vishnui*, *Cx. quinquefasciatus* Say, *An. minimus* s.l. Theobald, * An. maculatus* s.l. Theobald) represent important disease vectors in Thailand. This study also shows significant differences in the capture efficiency of CDC-LT when placed indoors or outdoors and in different seasons. Our study thus provides important guidelines for more targeted future vector trapping studies on the Thai-Myanmar border, which is an important cross-border malaria transmission region in Thailand.

**Electronic supplementary material:**

The online version of this article (doi:10.1186/s13071-015-1225-3) contains supplementary material, which is available to authorized users.

## Background

Collection and speciation of adult mosquitoes is important in entomological studies monitoring disease vectors, especially in malaria endemic regions [1]. Field studies that aim at describing the interactions of vectors with pathogens, human hosts and the environment help to understand region-specific transmission dynamics and disease spread, and guide efforts to control and eliminate diseases. While malaria is now absent from large parts of Thailand, it is still an important health problem in some border regions, particularly the northwestern border region with Myanmar. Tak province, Northwestern of Thailand, had a malaria incidence rate of 11.7 cases per 1,000 people in 2013 [2]. Malaria elimination programs are expected to make 80 % of Thailand malaria-free by 2020 [3]. To achieve this goal, well-developed and continuous surveillance programs in the border regions with Cambodia and Myanmar are required in order to quantify and control cross-border malaria transmission [4]. In this context, entomological surveillance plays an important role.

Human landing catches (HLC) are the standard method to study vector bionomics, as this technique is focused on host-seeking mosquitoes that may represent the most relevant proportion of the mosquito population for disease transmission [5]. However, the HLC approach is ethically controversial, particularly as the study-related risk of infection of the exposed individuals cannot be completely abrogated [1]. Furthermore HLC are labor intensive and difficult to standardize due to variation in individual attractiveness to mosquitoes and variation in the experience of individuals performing HLC assays [1].

A variety of alternative sampling and analysis methods have previously been studied in terms of their field efficiency and applicability, which depends on local vector populations [6, 7]. Odor-baited entry traps are a practical alternative to HLC for adult collection and CDC-LT are one of the most widely-used type of trap, and can be used for indoor and outdoor collection [1]. Several previous studies have shown statistically significantly better trapping results with CO_2_-baited CDC-LT [8–10], especially for *Culex* species. Specifically, increasing levels of CO_2_ (dissipated either by the sublimation of dry ice or from CO_2_ cylinders), up to a threshold of approximately 500 mL/min have been observed to be related with better trapping efficiency and higher numbers of trapped mosquitoes [11]. However, other studies have shown no difference in trapping efficiency for *Anopheles* adult female mosquitoes [12–14]. These mixed results and the fact that there have been very few studies on the effectiveness of CDC-LT in Thailand [14] warrant further investigations, especially since many previous studies lack multivariate analyses of factors that may influence trapping efficiency such as whether a trap was placed indoors or outdoors, geographic region, season of the year and feeding status of mosquitoes.

The present study was conducted in order to evaluate mosquito sampling by CDC-LT in 7 villages in northwestern Thailand and to analyze relative trap efficiency for traps augmented with CO_2_ baits and placed either indoors or outdoors. The region is one of the most malaria endemic areas in Thailand. There are typically two peaks in malaria case frequency, the first in the rainy season (May-July) and the second in the beginning of dry season during October to November [15]. The region is environmentally and demographically very diverse, endemic to both *Plasmodium falciparum* and *P. vivax* and home to a diversity of anopheline vector species making it a very complex malaria transmission environment. [4, 15–17]. This study was done mainly in order to identify the best conditions to trap specific potential vectors but also to further learn about the behavioral differences and preferences of different vectors of malaria and other vector-borne diseases and their distribution and abundance in this area. Our study shows important differences in trapping efficiency for different mosquito genera and species present in northwestern Thailand. The results are therefore useful for the planning of further, larger trapping studies and routine entomological surveillance programs, which would benefit from maximizing capture efficiency for potential disease vectors.

## Methods

### Study site

**Fig. 1:**
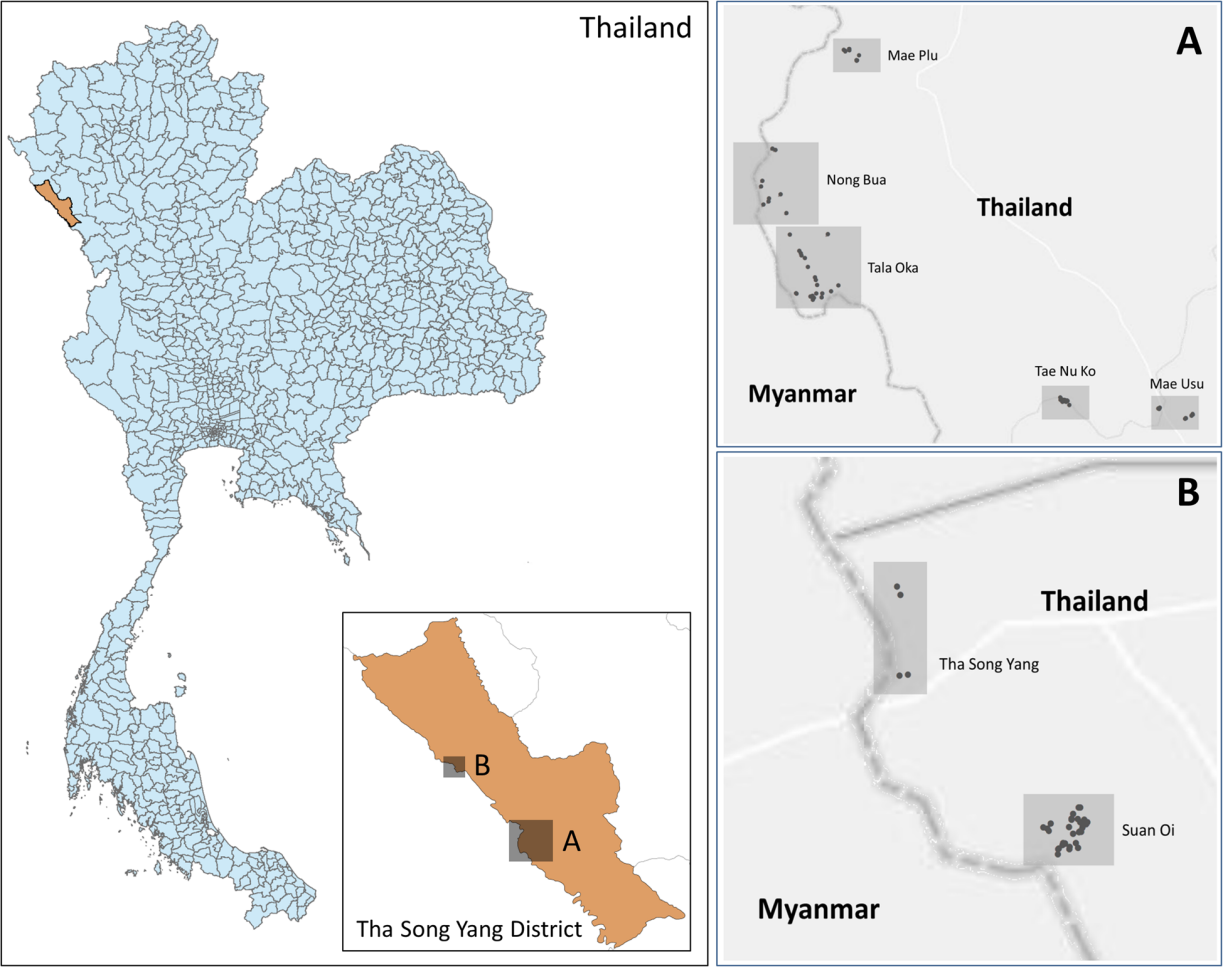
Map of the study sites. The left panels show the location of Tha Song Yang district, Tak province within Thailand and the location of the study regions (**a** and **b**). The right panels provide a more detailed view of the study sites. Mosquitoes were collected in 7 selected villages: Mae Usu, Tae Nu Ko, Mae Plu, Tha Song Yang, Suan Oi, Tala Oka and Nong Bua between Jan 2012 and Mar 2013. Data were aggregated by region (**a** and **b**)

Adult mosquitoes were collected using CDC-LT with and without CO_2_ baits in 7 villages in Tak Province, Thailand, located along the Thai-Myanmar border (Fig. 1), namely: Mae Usu, Tae Nu Ko, Mae Plu, Tha Song Yang, Suan Oi, Tala Oka and Nong Bua. Weather in the area is characterized by three seasons: hot (March to May), wet (June to August) and dry (September to February). Mean annual rainfall in the study period was 171.05 mm (range: 0 to 535 mm), mean annual temperature was 26.6 °C (range: 24.1-28.0 °C), and mean humidity was 72 % (range: 59-88 %). There are approximately 138,000 residents living in approximately 27,000 houses in these villages. Most people farm seasonal crops and rice, and engage in forestry work. The population is a mix of local Thai and either permanent or temporary Karen migrants from Myanmar. Most of the houses where traps were placed (*n* = 88) were located near a river and/or swamp areas, which are likely to represent mosquito breeding habitats.

According to Ministry of Public Health policy, vector control is being carried out in all active transmission areas, including the study area. Policy recommends that in-house residual spraying (IRS) is conducted twice a year in perennial transmission areas, and annually in periodic transmission areas covering the transmission season. In addition, permethrin insecticide treated nets (ITN) are distributed in high transmission areas and are offered (free of charge) by the malaria clinics. Thermal fogging is applied during malaria outbreaks once a week for 4 consecutive weeks. Among the selected houses, we found a surprisingly low actual ITN usage: only 50 % Thai houses and 30 % temporal Karen houses reported to use ITNs when asked before setting the traps.

### Study design and mosquito collection

**Fig. 2:**
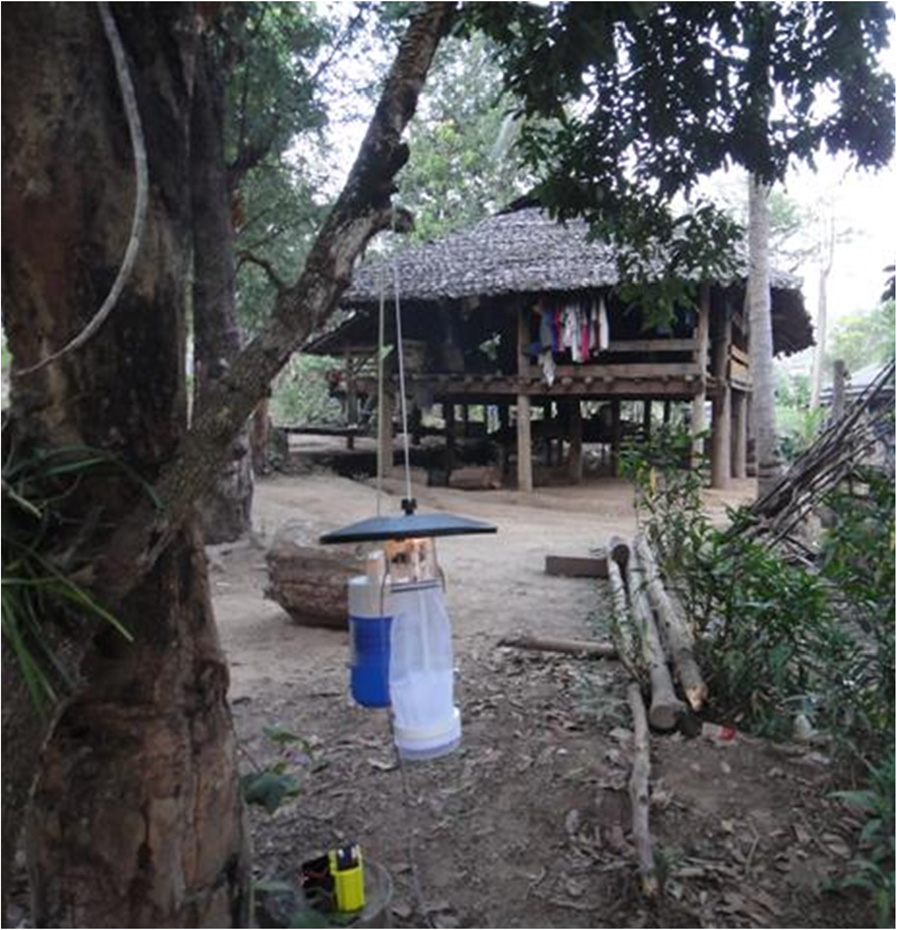
CDC light trap setting. The CDC light traps with and without 1 kg of dry ice were set by hanging them in about 1.5 meter height either indoors and outdoors (10–20 meters from house), overnight (6.00 pm.-6.00 am.). Mosquitoes were collected daily in the early morning

CDC-LT (*n* = 112, BioQuip model 2836BQ, with a 6 volt battery, USA) were placed inside or around 88 selected houses for stretches of five consecutive nights from January 2011 to March 2013. These stretches of 5 nights are from now on referred to as ‘trap placements’. There were a total of 576 trap placements corresponding to 2,880 trap-nights in the study period. Trap coverage varied between villages from 65 trap-nights in Tha Song Yang to 1,330 trap-nights in Suan Oi (Additional file 1: Table S2), and by season, from 55 trap-nights in February to 440 trap-nights in May. The traps were not placed in December. Because of this variation in sampling density, data were aggregated into 2 regions (north (A) and south (B) as indicated in Fig. 1) and into the 3 seasons (dry: September to February, hot: March to May and wet: June to August). The traps were installed by hanging them approximately 1.5 m above the ground either indoors (usually in the living room and some houses have only a single room) or outdoors (10–20 m away from houses, see Fig. 2). Approximately half of the traps (corresponding to a total of 1,600 trap-nights) were augmented with 1 kg of dry ice whereas the remaining traps (corresponding to a total of 1,280 trap nights) had no dry ice. The locations of the traps were recorded by GPS (Garmin GPSMAP 60CSx, USA). Mosquitoes were collected from the traps each morning and sent to the laboratory (Department of Medical Entomology, Faculty of Tropical Medicine, Mahidol University, Bangkok) for analysis. Mosquito species were determined based on morphological characteristics [18–21]. Mosquito blood meal status (empty, blood fed, half gravid, and gravid) was also recorded.

### Data analysis

Statistical analyses were performed in Stata 12 (StataCorp, USA). Absolute mosquito numbers were converted to mosquito capture rates, i.e., mosquitoes per trap per year (MTY). As the same traps were placed multiple times, multivariate analysis was conducted by multilevel negative binomial regression to account for overdispersion of the counts. Fixed effects were CO_2_ vs. no CO_2_, indoor vs. outdoor placement, region and season. Analyses were repeated for each relevant mosquito genus (*Culex, Anopheles, Armigeres, Aedes*) and species (*An. minimus* s.l., *An. maculatus* s.l., *An. annularis* s.l. van der Wulp*, Cx. vishnui, Cx. quinquefasciatus, Ae. albopictus*), as well as for blood-fed mosquitoes only in cases where trapped mosquito numbers allowed for this analysis. Effect measure modification was tested for by including interaction terms for trap placement (indoor/outdoor) and season as well as for trap type (CO_2_/non-CO_2_) and season.

## Results

### Mosquito trapping

**Fig. 3:**
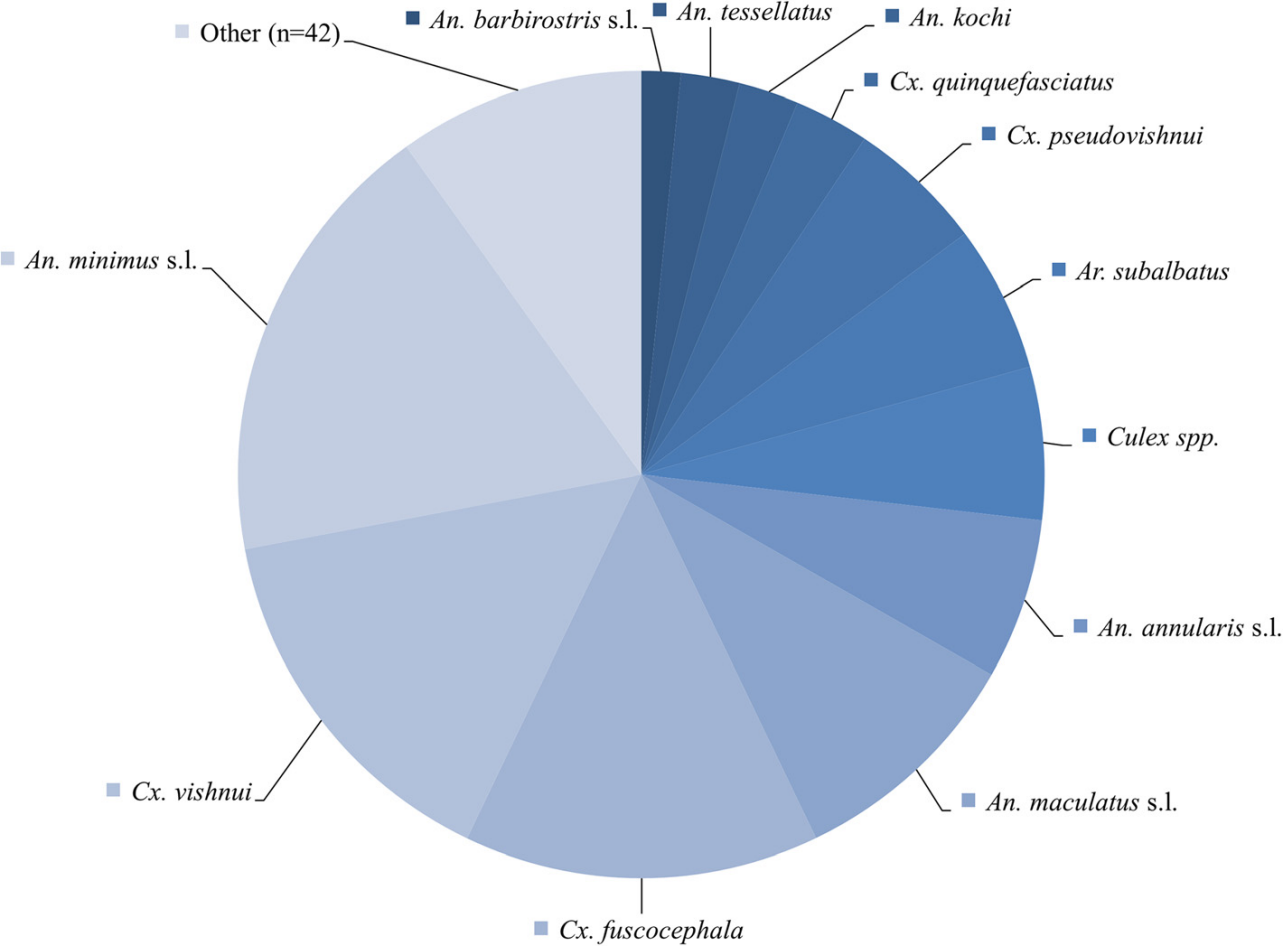
Schematic representation of the mosquitoes captured in the present study. Species that constituted less than 1 % of the total collected population were summarized as ‘other’. A full table of containing all mosquito species is given in the (Additional file 2: Table S1)

A total of 6,668 adult mosquitoes were collected in a total of 576 individual CDC-LT placements (2,880 trap-nights). A summary of the absolute numbers of mosquitoes captured per trap-type and indoor vs. outdoor placement is given in Table 1 and an overview over the most abundant mosquito species captured in this study is shown in Fig. 3. There were 42 species that represented less than 1 % of the total captured population and these are summarized as ‘others’. A table showing all capture data, including minority species, mosquito feeding and gravidity status, is presented in the (Additional file 2: Table S1). *Culex* spp. were predominant (46 % of the total collected mosquitoes were *Culex* spp.). The main *Culex* species were *Cx. vishnui* (*n* = 977)*, Cx. fuscocephala* Theobald (*n* = 951)*, Cx. pseudovishnui* Colless (*n* = 366)*,* and *Cx. quinquefasciatus* (*n* = 201) (Fig. 3). Forty-five percent (45 %) of captured mosquitoes were *Anopheles* spp. with *An. minimus* s.l. (*n* = 1206), *An. maculatus* s.l*.* (*n* = 641), and *An. annularis* s.l. (*n* = 431) as the most abundant species (Table 1, Fig. 3, Additional file 2: Table S1). Other important genera were *Armigeres* spp. (*n* = 404) where *Ar. subalbatus* (Coquillett) (*n* = 392) represented the vast majority and *Aedes* spp. mosquitoes with *Ae. albopictus* (*n* = 68) and *Ae. aegypti* (Linneaus) (*n* = 31) as the main representatives.

**Table 1:** Numbers of adult mosquitoes collected indoors and outdoors and using CDC-LT with and without CO_2_

Genus	LT with CO_2_		Sum (CO_2_)	LT without CO_2_		Sum (w/o CO_2_)	Total	% total
	Indoor	Outdoor		Indoor	Outdoor			
*Culex*	851	1120	1971	823	299	1122	3093	46.39
*Anopheles*	843	988	1831	784	373	1157	2988	44.81
*Armigeres*	73	164	237	101	58	159	396	5.94
*Aedes*	43	46	89	52	19	71	160	2.40
*Uranotaenia*	1	2	3	7	3	10	13	0.19
*Mansonia*	4	0	4	4	0	4	8	0.12
*Topomyia*	1	0	1	3	1	4	5	0.07
*Ficalbia*	0	0	0	0	3	3	3	0.04
*Aedeomyia*	0	0	0	2	0	2	2	0.03
Total	1816	2320	4136	1776	756	2532	6668

Most of the collected mosquitoes were female (94.4 %) and in the empty stage (94.0 %). The ratio of blood-fed mosquitoes varied between species with *Cx. vishnui* (1.3 %), *Cx. quinquefasciatus* (1.5 %), *An. minimus* s.l. (13 %), *An. maculatus* s.l. (7 %), *An. annularis* s.l. (4 %), *Ae. aegypti* (50 %), and *Ae. albopictus* (8 %) collected as blood-feds. Table 2 shows the multivariate analysis results of mosquito capture rate for the main vector genera and species detected in this study.

**Table 2:** Numbers of mosquitoes trapped by CDC-LT, and CDC-LT performance

	Trap-nights	n	MTY	aCRR	P	95 % CI	
***Ae. albopictus*** **(** ***n*** **= 68)**							
Trap w/o CO_2_ (reference)	1280	36	10				
Trap with CO_2_	1600	32	7	0.81	0.52	0.41	1.56
Indoor trap (reference)	1655	51	11	-	-	-	-
Outdoor trap	1225	17	5	0.53	0.07	0.27	1.05
***Anopheles*** **spp. (** ***n*** **= 2989)**							
Trap w/o CO_2_ (reference)	1280	1157	330				
Trap with CO_2_	1600	1832	418	**1.39**	**0.04**	**1.01**	**1.61**
Indoor trap (reference)	1655	1628	359	-	-	-	-
Outdoor trap	1225	1361	406	0.92	0.53	0.74	1.17
***An. annularis*** **s.l. (** ***n*** **= 431)**							
Trap w/o CO_2_ (reference)	1280	171	49				
Trap with CO_2_	1600	260	59	1.28	0.37	0.75	2.17
Indoor trap (reference)	1655	161	35	-	-	-	-
Outdoor trap	1225	270	80	1.4	0.21	0.83	2.34
***An. maculatus*** **s.l. (** ***n*** **= 641)**							
Trap w/o CO_2_ (reference)	1280	250	71	**-**	**-**	**-**	**-**
Trap with CO_2_	1600	391	89	1.27	0.18	0.90	1.75
Indoor trap (reference)	1655	378	83	**-**	**-**	**-**	**-**
Outdoor trap	1225	263	78	0.52	0.60	0.79	1.51
***An. minimus*** **s.l. (** ***n*** **= 1206)**							
Trap w/o CO_2_ (reference)	1280	448	128				
Trap with CO_2_	1600	758	173	1.27	0.11	0.95	1.69
Indoor trap (reference)	1655	679	150	-	-	-	-
Outdoor trap	1225	527	157	**0.71**	**0.02**	**0.52**	**0.95**
***Armigeres spp.*** **(** ***n*** **= 404)**							
Trap w/o CO_2_ (reference)	1280	159	45				
Trap with CO_2_	1600	245	55	1.22	0.28	0.89	1.75
Indoor trap (reference)	1655	174	38	-	-	-	-
Outdoor trap	1225	230	68	**1.5**	**0.02**	**1.06**	**2.12**
***Culex spp.*** **(** ***n*** **= 3094)**							
Trap w/o CO_2_ (reference)	1280	1122	320				
Trap with CO_2_	1600	1972	450	1.16	0.23	0.91	1.49
Indoor trap (reference)	1655	1675	369	-	-	-	-
Outdoor trap	1225	1419	423	0.85	0.19	0.66	1.08
***Cx. quinquefasciatus*** **(** ***n*** **= 201)**							
Trap w/o CO_2_ (reference)	1280	53	15				
Trap with CO_2_	1600	148	34	1.02	0.94	0.61	1.72
Indoor trap (reference)	1655	157	32	-	-	-	-
Outdoor trap	1225	44	13	0.67	0.14	0.39	1.13
***Cx. vishnui*** **(** ***n*** **= 997)**							
Trap w/o CO_2_ (reference)	1280	280	250				
Trap with CO_2_	1600	717	391	**1.64**	**0.02**	**1.09**	**2.44**
Indoor trap (reference)	1655	446	378	-	-	-	-
Outdoor trap	1225	531	263	0.88	0.55	0.59	1.32

Note: n: absolute number of mosquitoes captured; MTY: mosquitoes per trap and year; aCRR: adjusted capture rate ratio; 95 % CI: 95 % confidence interval; Bold numbers: significant difference in aCRR (*P* < 0.05). *Ae.: Aedes, An.: Anopheles, Cx.: Culex,* w/o: with/without

Comparisons were made between traps augmented with CO_2_ baits and those with no baits and between traps placed in indoor and outdoor locations. Models are adjusted for region and season

*Anopheles* spp. mosquitoes tended to be captured more efficiently in CO_2_-baited traps (adjusted capture rate ratio (aCRR) 1.39, *P* = 0.04). This trend became statistically insignificant when the main *Anopheles* species were analyzed separately (*An. minimus* s.l. aCRR: 1.27, *P* = 0.11; *An. maculatus* s.l. aCRR: 1.28, *P* = 0.18, *An. annularis* s.l. aCRR: 1.28, *P* = 0.37). In addition, *An. minimus* s.l. was captured more frequently indoors (aCRR for outdoors: 0.71, *P* = 0.02).

Overall, *Culex* species were trapped in the CO_2_ baited traps with similar efficiency as in the non-baited traps. However, there was a significant difference when the analysis was restricted to *Cx. vishnui* (aCRR: 1.64, *P* = 0.02). *Armigeres* spp. mosquitoes were captured more frequently outdoors (aCRR: 1.5, *P* = 0.02) whereas *Ae. albopictus* tended to be captured more frequently indoors (aCRR for outdoor traps 0.53, *p* = 0.07). No differences in these trends were found when only the blood-fed mosquitoes were analyzed.

### Effect measure modification by indoor vs. outdoor placement

Whether the traps were placed inside houses or outdoors played a significant role. CO_2_ baited traps performed better for anopheline mosquitoes than unbaited traps in outdoor locations (aCRR 1.56, *P* = 0.02) but performed similarly well in indoor locations (aCRR: 1.14, *P* = 0.41). This may be explained by the presence of occupants indoors representing bait and thus enhancing the efficiency of CDC-LT even without CO_2_. Similarly, *Culex* mosquitoes had a tendency to be captured better in the CO_2_ baited traps than in the unbaited traps in outdoor locations (aCRR 1.49; *P* = 0.07), while the indoor baited traps performed similar to the indoor non-baited traps (aCRR 1.01; *P* = 0.96) and thus the overall effect was not significant (Table 2). *Armigeres* spp. were also captured significantly better in CO_2_ baited traps placed outdoors than in the non-baited traps placed outdoors (aCRR 2.17, *P* = 0.01), but tended to be captured less well in the CO_2_ baited traps placed indoors (aCRR 0.71; *P* = 0.10).

### Effect measure modification by seasonality

We have previously shown strong, species-specific seasonality in mosquito abundance in this region [22]. Therefore, effect measure modification of trapping efficacy by seasonality was examined. Better capture rate of *Anopheles* spp. mosquitoes by CO_2_ baited traps was restricted to the hot season (March to May, aCRR 1.49, *p* = 0.04), whereas in the other seasons (rainy and dry), CO_2_ baited traps were equivalent to non-baited traps (*P* = 0.30 and *P* = 0.88, respectively). Seasonality did not vary the capture rate difference between indoor and outdoor traps for *Anopheles* spp. These observations extended to *An. minimus* s.l.. The other *Anopheles* species were not abundant enough to fit the interaction model.

Similarly, the effect of better outdoor trapping of *Culex* spp. in CO_2_ baited traps was restricted to the hot season (effect measure modification 1.94, *P* = 0.05). These observations did not hold when only *Cx. vishnui* was considered. *Armigeres* spp. mosquitoes were captured consistently better outdoors and this effect was not modified by seasonality. No effect measure modification was observed for *Aedes* spp. mosquitoes.

## Discussion

This study evaluated the efficiency of CDC-LT used with or without CO_2_ baits and placed inside or outside of residential dwellings in northwestern Thailand. This is the first in-depth survey and analysis, seeking to provide some guidelines for CDC-LT-based mosquito trapping studies and surveillance programs in this region of Thailand.

Overall, CO_2_ baits significantly increased trapping efficiency of *Anopheles* spp. mosquitoes (approximately 40 % observed increase in aCRR), especially when the traps were placed outside of residential dwellings. Stratification by season revealed that the effect was restricted to observations in the hot-season (March to May). Generally, the most abundant *Anopheles* species, *An. minimus* s.l. was captured preferentially in indoor traps, which is likely related to its anthropophilic nature [22]. We therefore conclude that CO_2_ baits are beneficial when targeting *Anopheles* spp., as their use may lead to increased capture rates in comparison to non-baited CDC-LT. These findings are consistent with previous studies, which have shown that dry ice baited CDC-LT are a good alternative choice to collect malaria vectors including *An. minimus* s.l.*,* and *An. maculatus* s.l. and *An. sawadwongporni* Rattanarithikul and Green*,* respectively [14, 23]. In contrast, previous studies on African and Brazilian malaria vectors, specifically *An. arabiensis* Patton, *An. funestus* s.l. Giles , *An. darlingi* Root , and *An. aquasalis* Curry have shown that CO_2_ was insufficiently attractive as a stand-alone bait and that traps using CO_2_ in mixed odor baits or together with body odors may provide better results [12, 24, 25]. Most of the collected *Anopheles* mosquitoes were in the unfed state and feeding status did not seem to impact capture efficiency when comparing indoor and outdoor trap locations. This stands in contrast to a previous study that indicated a preferential capture of blood-fed mosquitoes (*Anopheles quadriannulatus* (Theobald) and *An. funestus* s.l.) by CDC-LT in indoor locations in Zambia [26], however this may be attributable to the low numbers of blood fed mosquitoes observed in this study and that the captured *Anopheles* species commonly exhibit a zoophilic host preference [27].

*Culex* spp. were the most abundant species collected in this study. Overall, there was no significant difference in the capture efficiency of baited or unbaited traps and/or trap locations (Table 2). Similar to *Anopheles* spp. there was a tendency that CO_2_ baited traps were more efficient than unbaited traps in outdoor locations. When *Cx. vishnui* was considered separately, capture efficiency was significantly higher in CO_2_ baited traps. More detailed analysis revealed that this effect was restricted to traps placed outdoors and in the hot season (as compared to unbaited traps placed outdoors in the hot season).

*Cx. vishnui* is a main vector of Japanese Encephalitis Virus (JEV) [28, 29]. It is most commonly found in fragmented forest, rural, and suburban habitats and is exophagic in nature, preferentially feeding on pigs [30, 31]. This may explain why it is more frequently trapped in outdoor locations. Previous studies have shown improved collected mosquito numbers in CO_2_ baited traps for *Cx. quinquefasciatus* in French Polynesia [32], and *Cx. quinquefasciatus* and *Cx. annulioris* Theobald in Kenya [13]. In addition, the use of CDC-LT with dry ice was most effective for trapping of *Cx. quinquefasciatus* when compared with UV light traps and gravid traps in China [33]. This effect was not observed in the present study but this may be attributable to the low numbers of *Cx. quinquefasciatus* captured. Traps were mostly placed in villages surrounded by mountains and forests whereas *Cx. quinquefasciatus* is a mostly urban mosquito species and known to breed in open drains polluted with organic matter [20, 34]. Therefore, the trap setting strategy applied in this study may not have been suitable to capture large numbers of *Cx. quinquifasciatus.*

*Armigeres* mosquitoes (>95 % *Ar. subalbatus*) were captured consistently better outdoors in the CO_2_ baited traps and this effect was consistent across seasons. *Ar. subalbatus* primarily occurs in plantation areas and forests, and is mainly active during the day particularly in the crepuscular period [18]. This may explain its preferential capture in outdoor locations. *Ar. subalbatus* is known to transmit *Wuchereria bancrofti* and several zoonotic filarial worms such as *Brugia pahangi* [35, 36]. While some previous studies have compared captured *Ar. subalbatus* numbers using different types of traps, we are not aware of a direct comparison of CO_2_ vs. non-CO_2_ and indoor vs. outdoor trap placements for this mosquito species [37].

Overall, the number of *Aedes* species mosquitoes captured in this study was low and most captured *Aedes* mosquitoes were *Ae. albopictus*. Although previous studies have indicated that CDC-LT are amongst the most efficient traps for the capture of some *Aedes* species [38] these differences were not apparent for *Ae. albopictus*. CO_2_ baiting slightly increased *Ae. aegypti* capture in a comparative trapping study in Manaus [39]. In the present study, there were no statistically significant differences in trapping efficacy with or without CO_2_ and the placement of the traps. *Ae. albopictus* seemed to have a tendency of preferential indoor capture (*P* = 0.07). Extended trapping studies would need to be conducted in order to determine whether capture efficiency is improved by CO_2_ and/or whether indoor/outdoor trap placement is important. *Aedes* trapping studies commonly use BG traps and it has been shown that these are more effective in capturing *Aedes* than CDC-LT [40, 41].

This study is limited by several factors. Trap placement was irregular and the number of trap nights differed considerably between villages and months of year (see Additional file 1: Tables S2 and Additional file 3: Table S3). While most previous studies distinguish between traps by counting absolute mosquito numbers, due to the complex and irregular placement of the traps in this study we compared the rate of mosquito capture per unit time, rather than absolute numbers [14, 39]. Although CDC-LT baited with CO_2_ were shown to increase capture rate for several mosquito species including several important disease vectors (*Anopheles* spp*., Cx. vishnui, Ar. subalbatus*), it should be noted that the traps require daily dry ice and battery changes limiting the scope of trapping studies, as each trap needs to be maintained every day. Over 94 % of female mosquitoes in the trapped population were not blood-fed. It is unclear whether these individuals are newly emerged (nulliparous) or parous females that have not yet taken a blood meal. The ratio of nulliparous to parous female mosquitoes (determined e.g., by dissection) may represent an important entomological parameter to be determined in future studies. Normally, *An. minimus* s.l. and *An. maculatus* s.l. are regarded as exophilic [42]. A surprisingly small percentage of occupants in the study houses reported using ITNs (40-50 % Thai and 30 % Karen).

We cannot exclude the possibility that concurrent usage of ITN decreased indoor biting, but our analyses did not show such an effect modification, possibly because our sample numbers are too small. Other factors, such as house structures and the presence of domestic animals around houses might further affect mosquito behavior.

Further studies should be conducted to comparatively evaluate whether the species composition, and the blood-fed and physiological age distribution of captured mosquitoes is similar for CDC-LT and human landing catches and thus, if CDC-LT are truly capable of capturing representative samples of those mosquitoes relevant for human disease transmission. This study highlights differences in trapping efficiency of CDC-LT (baited and unbaited) for different mosquito species. Our study thus provides important orientation for more targeted future vector trapping studies on the Thai-Myanmar border, an important cross-border malaria transmission region.

## Conclusion

The present study shows that CDC-LT baited with CO_2_ generally capture more *Anopheles*, *Culex* and *Armigeres* mosquitoes than unbaited traps, especially when the traps are placed in outdoor locations. When traps were placed in indoor locations, there was little or no difference in baited vs. unbaited CDC-LT. Comparative trapping efficacy also varies with season. The results of the present study provide guidance for future entomological studies for surveillance of the local mosquito vectors in northwestern Thailand and elsewhere.

## Additional files

Additional file 1: Table S2. Trap-nights and mosquitoes caught per trap and year in each of the seven villages for the most abundant *Anopheles* mosquito species. (DOCX 25 kb) Additional file 2: Table S1. All mosquitoes captured in the present study, their blood-fed status, percentage of females and gravidity status. (DOCX 18 kb) Additional file 3: Table S3. Trap-nights and mosquitoes caught per trap and year in each of the seven villages for the most abundant mosquito genera and species other than *Anopheles*. (DOCX 19 kb)
