# Supplementary material for: Evaluation of CDC light traps for mosquito surveillance in a malaria endemic area on the Thai-Myanmar border
Source: Parasit Vectors. 2015 Dec 15;8:636. doi: 10.1186/s13071-015-1225-3 (PMC4678759; doi:10.1186/s13071-015-1225-3)
Supplement: Additional file 1: Table S2. — Trap-nights and mosquitoes caught per trap and year in each of the seven villages for the most abundant Anopheles mosquito species. (DOCX 25 kb) [file 13071_2015_1225_MOESM1_ESM.docx]

# Supporting Information Tables

**Table S2: Trap-nights and mosquitoes caught per trap and year in each of the seven villages for the most abundant *Anopheles* mosquito species.**

| Village | Trap-nights | n | *Mosquitoes/*  *TrapYear* |
| --- | --- | --- | --- |
| ***Anopheles spp.* (n=2,989)** |  |  |  |
| Mae Plu | 140 | 76 | 198 |
| Mae Usu | 145 | 31 | 78 |
| Nong Bua | 360 | 235 | 238 |
| Suan Oi | 1,330 | 966 | 265 |
| Tae Nu Ko | 270 | 80 | 108 |
| Tala Oka | 735 | 1,698 | 843 |
| Tha Song Yang | 65 | 8 | 44 |
|  |  |  |  |
| ***An. annularis* s.l. *(n=431)*** |  |  |  |
| Mae Plu | 140 | 0 | 0 |
| Mae Usu | 145 | 0 | 0 |
| Nong Bua | 360 | 51 | 51.7 |
| Suan Oi | 1,330 | 15 | 4.1 |
| Tae Nu Ko | 270 | 0 | 0 |
| Tala Oka | 735 | 365 | 181.3 |
| Tha Song Yang | 65 | 0 | 0 |
|  |  |  |  |
| ***An. maculatus* s.l. *(n=641)*** |  |  |  |
| Mae Plu | 140 | 3 | 7.8 |
| Mae Usu | 145 | 3 | 7.6 |
| Nong Bua | 360 | 63 | 63.9 |
| Suan Oi | 1,330 | 286 | 78.5 |
| Tae Nu Ko | 270 | 7 | 9.5 |
| Tala Oka | 735 | 295 | 146.5 |
| Tha Song Yang | 65 | 1 | 5.6 |

| ***An. minimus* s.l. *(n=1,206)*** |  |  |  |
| --- | --- | --- | --- |
| Mae Plu | 140 | 62 | 162 |
| Mae Usu | 145 | 24 | 60 |
| Nong Bua | 360 | 71 | 72 |
| Suan Oi | 1,330 | 512 | 141 |
| Tae Nu Ko | 270 | 29 | 39 |
| Tala Oka | 735 | 556 | 276 |
| Tha Song Yang | 65 | 6 | 34 |

# Note: n: absolute number of mosquitoes captured. *An.: Anopheles.*
