# Supplementary material for: Evaluation of CDC light traps for mosquito surveillance in a malaria endemic area on the Thai-Myanmar border
Source: Parasit Vectors. 2015 Dec 15;8:636. doi: 10.1186/s13071-015-1225-3 (PMC4678759; doi:10.1186/s13071-015-1225-3)
Supplement: Additional file 2: Table S1. — All mosquitoes captured in the present study, their blood-fed status, percentage of females and gravidity status. (DOCX 18 kb) [file 13071_2015_1225_MOESM2_ESM.docx]

# Supporting Information Tables

Table S1: All mosquitoes captured in the present study, their blood-fed status, percentage of females and gravidity status.

| **Species/Genus** | **n** | **Blood-fed (%)** | **Female (%)** | **Gravid (%)** |
| --- | --- | --- | --- | --- |
| *Ad. catasticta* | 2 | 0 (0) | 2 (100) | 0 (0) |
| *Ae. aegypti* | 31 | 16 (51.6) | 29 (93.5) | 1 (3.22) |
| *Ae. albopictus* | 68 | 6 (8.82) | 55 (80.8) | 1 (1.47) |
| *Ae. desmotes* | 4 | 1 (25) | 3 (75) | 0 (0) |
| *Ae. mucidus* | 1 | 0 (0) | 1 (100) | 0 (0) |
| *Ae. vexans* | 32 | 1 (3.12) | 32 (100) | 0 (0) |
| *Aedes* spp. | 14 | 1 (7.14) | 13 (92.8) | 0 (0) |
| *An. aconitus* | 2 | 0 (0.00) | 2 (100) | 0 (0) |
| *An. annularis* s.l. | 431 | 18 (4.17) | 431 (100) | 3 (0.69) |
| *An. barbirostris* s.l. | 127 | 16 (12.59) | 127 (100) | 1 (0.79) |
| *An. culicifacies* s.l. | 51 | 14 (27.4) | 51 (100) | 0 (0) |
| *An. dirus* s.l*.* | 17 | 3 (17.6) | 17 (100) | 0 (0) |
| *An. dravidicus* | 3 | 0 (0) | 3 (100) | 0 (0) |
| *An. indefinitus* | 7 | 1 (14.2) | 7 (100) | 0 (0) |
| *An. jamesii* | 18 | 1 (5.55) | 18 (100) | 0 (0) |
| *An. kochi* | 161 | 10 (6.21) | 161 (100) | 0 (0) |
| *An. maculatus* s.l. | 645 | 45 (7.02) | 627 (97.2) | 0 (0) |
| *An. minimus* s.l. | 1,206 | 166 (13.7) | 1,203 (99.7) | 0 (0) |
| *An. nigerrimus* | 9 | 0 (0) | 9 (100) | 0 (0) |
| *An. nivipes* | 3 | 0 (0) | 3 (100) | 0 (0) |
| *An. peditaeniatus* | 63 | 8 (12.6) | 63 (100) | 0 (0) |
| *An. philippinensis* | 8 | 1 (12.5) | 8 (100) | 0 (0) |
| *An. pseudojamesii* | 20 | 0 (0) | 20 (100) | 0 (0) |
| *An. pseudowillmori* | 1 | 0 (0) | 1 (100) | 0 (0) |
| *An. sawadwongporni* s.l. | 4 | 0 (0) | 4 (100) | 0 (0) |
| *An. sinensis* | 1 | 0 (0) | 1 (100) | 0 (0) |
| *An. subpictus* s.l. | 4 | 1 (25) | 4 (100) | 0 (0) |
| *An. tessellatus* | 157 | 1 (0.63) | 157 (100) | 0 (0) |
| *An. vagus* | 13 | 1 (7.69) | 13 (100) | 0 (0) |
| *An. varuna* | 41 | 9 (21.9) | 41 (100) | 0 (0) |
| *Ar. magnus* | 6 | 1 (16.6) | 6 (100) | 0 (0) |
| *Ar. malayi* | 4 | 0 (0) | 3 (75) | 0 (0) |
| *Ar. subalbatus* | 392 | 14 (3.57) | 352 (89.7) | 7 (1.78) |
| *Armigeres* spp. | 2 | 0 (0) | 2 (100) | 0 (0) |
| *Cx. bitaeniorhynchus* | 56 | 1 (1.78) | 56 (100) | 0 (0) |
| *Cx. fuscocephala* | 951 | 8 (0.84) | 739 (77.7) | 19 (1.99) |
| *Cx. gelidus* | 12 | 1 (8.33) | 12 (100) | 0 (0) |
| *Cx. hutchinson* | 10 | 0 (0) | 10 (100) | 0 (0) |
| *Cx. pseudovishnui* | 366 | 15 (4.09) | 358 (97.8) | 3 (0.81) |
| *Cx. quinquefasciatus* | 201 | 3 (1.49) | 147 (73.1) | 5 (2.48) |
| *Cx. sinensis* | 1 | 0 (0) | 0 (0) | 0 (0) |
| *Cx. sitiens* | 3 | 0 (0) | 3 (100) | 0 (0) |
| *Cx. tritaeniorhynchus* | 76 | 11 (14.4) | 73 (96.0) | 0 (0) |
| *Cx. vishnui* | 997 | 13 (1.30) | 987 (98.9) | 2 (0.20) |
| *Cx. whitmorei* | 13 | 0 (0) | 13 (100) | 0 (0) |
| *Culex* spp. | 408 | 5 (1.22) | 404 (99.0) | 0 (0) |
| *Downsiomyia* spp. | 9 | 4 (44.4) | 9 (100) | 0 (0) |
| *Ficalbia* spp. | 3 | 0 (0) | 3 (100) | 0 (0) |
| *Ma. uniformis* | 8 | 1 (12.5) | 8 (100) | 0 (0) |
| *Topomyia* spp. | 5 | 0 (0) | 5 (100) | 0 (0) |
| *Uranotaenia* spp. | 13 | 1 (7.69) | 10 (76.9) | 0 (0) |
| ***Total*** | **6,668** | **395 (5.96)** | **6,297 (94.4)** | **42 (0.62)** |

# Note: n: absolute number of mosquitoes captured; *Ad.* : *Aedeomyi; Ae. : Aedes; An.: Anopheles; Ar.; Armigeres; Cx. : Culex; Ma. : Mansonia.*
