# Supplementary material for: Evaluation of CDC light traps for mosquito surveillance in a malaria endemic area on the Thai-Myanmar border
Source: Parasit Vectors. 2015 Dec 15;8:636. doi: 10.1186/s13071-015-1225-3 (PMC4678759; doi:10.1186/s13071-015-1225-3)
Supplement: Additional file 3: Table S3. — Trap-nights and mosquitoes caught per trap and year in each of the seven villages for the most abundant mosquito genera and species other than Anopheles. (DOCX 19 kb) [file 13071_2015_1225_MOESM3_ESM.docx]

# Supporting Information Tables

**Table S3: Trap-nights and mosquitoes caught per trap and year in each of the seven villages for the most abundant mosquito genera and species other than *Anopheles*.**

| Village | Trap-nights | n | *Mosquitoes/*  *Trap-Year* |
| --- | --- | --- | --- |
| *Armigeres* spp. *(n=404)* |  |  |  |
| Mae Plu | 140 | 31 | 81 |
| Mae Usu | 145 | 21 | 53 |
| Nong Bua | 360 | 57 | 58 |
| Suan Oi | 1,330 | 177 | 49 |
| Tae Nu Ko | 270 | 36 | 49 |
| Tala Oka | 735 | 68 | 34 |
| Tha Song Yang | 65 | 14 | 79 |
|  |  |  |  |
| *Cx. quinquefasciatus (n=201)* |  |  |  |
| Mae Plu | 140 | 4 | 10 |
| Mae Usu | 145 | 22 | 55 |
| Nong Bua | 360 | 37 | 38 |
| Suan Oi | 1,330 | 49 | 13 |
| Tae Nu Ko | 270 | 51 | 69 |
| Tala Oka | 735 | 100 | 50 |
| Tha Song Yang | 65 | 1 | 6 |
|  |  |  |  |
| *Cx. vishnui (n=997)* |  |  |  |
| Mae Plu | 140 | 1 | 3 |
| Mae Usu | 145 | 13 | 3 |
| Nong Bua | 360 | 84 | 63 |
| Suan Oi | 1,330 | 121 | 286 |
| Tae Nu Ko | 270 | 26 | 7 |
| Tala Oka | 735 | 769 | 295 |
| Tha Song Yang | 65 | 4 | 1 |
|  |  |  |  |

# Note: n: absolute number of mosquitoes captured. *Cx.: Culex.*
